# Calcium/Calmodulin-Dependent Kinases in the Hypothalamus, Pituitary, and Pineal Gland: An Overview

**DOI:** 10.1155/2022/1103346

**Published:** 2022-12-26

**Authors:** Vincenzo Cimini, Susan Van Noorden, Cristina Terlizzi, Giovanna Giuseppina Altobelli

**Affiliations:** ^1^Department of Advanced Biomedical Sciences, Medical School, “Federico II” University of Naples, Naples, Italy; ^2^Department of Histopathology, Imperial College London, Hammersmith Hospital, London, UK

## Abstract

We review the literature on the little-known roles of specific CaMKs in regulating endocrine functions of the pineal gland, the pituitary gland, and the hypothalamus. Melatonin activates hippocampal CaMKII, which then influences dendritogenesis. In the pituitary gland, the signal pathways activated by the CaMK in lower vertebrates, such as fishes, differ from those of mammals. In the teleost anterior pituitary, the activation of CaMKII induces the expression of somatolactin by glucagon b. In rats and humans, CaMKIVs have been associated with gonadotropes and thyrotropes and CaMKII with several types of human tumor cells and with a specific signaling pathway. Neuropeptides such as vasopressin and endothelin are also involved in the CaMKII signaling chain, as is the CaMKII*δ* isoform which participates in generating the circadian rhythms of the suprachiasmatic nucleus. What arises from this review is that most of the hypothalamic CaMKs are involved in activities of the endocrine brain. Furthermore, among the CaMKs, type II occurs with the highest frequency followed by CaMKIV and CaMKI.

## 1. Introduction

The calcium ion (Ca^2+^) is a second messenger widely used in numerous cellular processes [1]. Increases of cytoplasmic Ca^2+^ concentrations, due to either internal or external sources, activate different cellular events and responses such as cell proliferation, gene transcription, muscle contraction, neurotransmitter release, exocytosis, apoptosis, and cell-cell communication [2, 3]. Cells have developed many mechanisms for maintaining homeostasis of Ca^2+^ and are able to retain it in low intracellular concentrations. Recent reports have demonstrated that intracellular Ca^2+^ receptors such as calretinin, calbindin, and other cytosolic EF-hand Ca^2+^-binding proteins are Ca^2+^-buffering substances that can also regulate signaling cascades [4]. Ca^2+^ destabilization, for example induced by the treatment of the hippocampus with *β* amyloid, is supposed to make the calretinin-containing neurons less vulnerable following the *β* amyloid-caused increase of its expression [5, 6]. However, the major intracellular EF-hand Ca^2+^ receptor is calmodulin (CaM), a small, highly conserved Ca^2+^ sensor able to activate downstream target enzymes. It functions as a receptor to sense changes in cellular Ca^2+^ concentration, and in this way, it mediates the role of the second messenger of this ion. Ca^2+^ binding to CaM induces a structural modification and the formation of a Ca^2+^/calmodulin complex [7] that enables CaM to interact with many target proteins leading to a decline in the concentration of Ca^2+^ ions. So, Ca^2+^ binds to CaM through the EF-hand structural motif and, when four of these binding sites are filled, CaM undergoes a conformational change becoming loaded with calcium and capable of interacting with one of its main targets in the cell. Among the several target proteins, there is a family of protein kinases called multifunctional calmodulin-dependent kinases (CaMKs) that includes CaMKI, CaMKII, CaMKIV, and CaMK kinase (CaMKK) that can act on many downstream target proteins [8, 9]. Another CaMK family can phosphorylate a single specific substrate such as myosin light chain kinase (MLCK). The CaMKs react rapidly to the increase in intracellular calcium due to the formation of the Ca^2+^/CaM complex, which in turn binds to kinase.

CaMKs, activated by increases of Ca^2+^ levels and by binding Ca^2+^/CaM, can regulate proteins by phosphorylation. They are widely expressed in different cell types and play a key role in numerous cellular processes such as neuronal functioning, learning, memory, gene transcription/expression, and cell proliferation [10].

In addition to differences in activity, CaMKs differ in tissue distribution and also in subcellular localization (Table 1). While CaMKIV is tissue-specific, CaMKI and CaMKII are ubiquitous [12].

There is considerable evidence that Ca^2+^ regulates hormone secretion in endocrine cells [13–16] and that increase of intracellular Ca^2+^ initiates signaling cascades that lead to essential biological processes such as secretion. It is still not clear which endocrine brain cell types express which CaMKs; therefore, the focus of this article, after a brief description of general aspects of CaMKs, will be to review the key data that have revealed the presence of specific CaMK isoforms and have suggested their possible role in the endocrine hypothalamus and brain endocrine glands.

## 2. CaMKs: Transcription Regulation

The intracellular concentration of Ca^2+^ is kept lower than that of the extracellular environment, thanks to numerous control mechanisms, and increases following various stimuli. Tyrosine kinase receptors and G protein-coupled receptors promote an increase of Ca^2+^ in the cytoplasm through the formation of inositol triphosphate (IP3). This induces the release of Ca^2+^ from intracellular deposits, the cisternae of the endoplasmic reticulum. The ligand-dependent and voltage-dependent Ca^2+^ channels present in the membrane, on the other hand, promote the entry of Ca^2+^ into the cell from the extracellular environment [17].

A central autoinhibitory region of the inactive kinase becomes activated only when bound to Ca^2+^/CaM; otherwise, it is intrasterically inhibited. Following binding, the enzyme starts its activity that is specific for each CaMK [18]. CaMKs are composed of an *N*-terminal catalytic domain and a central regulatory domain. The latter domain contains both an autoinhibitory region, which avoids substrate binding and a CaM binding domain.

### 2.1. CaMKI

CaMKI activation, as for CaMKII and CaMKIV, occurs by binding to Ca^2+^/CaM and is followed by auto threonine 177-phosphorylation in the activation loop. This event increases the enzymatic activity of the kinases and gives them greater substrate specificity [8].

CaMKI is expressed in the cytoplasm of mammalian cells. It has no canonical nuclear localization sequence (NLS), and it is not yet clear whether it can translocate into the nucleus; however, the existence of a nuclear isoform containing NLS has been demonstrated in *Caenorhabditis elegans* [19, 20].

### 2.2. CaMKII

CaMKII exists in different isoforms, expressed differently in the various cell types depending on which of the four genes encoding the kinase is expressed (*α*, *β*, *γ*, and *δ*) and the variants of “splicing.” The Ca^2+^/CaM complex is activated by binding to the kinase subunit, which self-transphosphorylates in threonine 286 in a hydrophobic pocket that works as a catalytic domain. The autophosphorylation occurs between two adjacent subunits and requires the binding of Ca^2+^/CaM to both the kinase subunit and the substrate subunit. This event prolongs the activity of the enzyme in two ways: first, the affinity of the enzyme for Ca^2+^/CaM increases in a process called “CaM trapping,” in which CaM remains trapped in the enzyme complex until the cytosolic Ca^2+^ returns to baseline levels; second, an autoinhibitory domain is displaced by converting the enzyme into a form independent of Ca^2+^, so that the kinase still has residual activity even after the initial activator signal has ended. Dephosphorylation of threonine 286 leads to inactivation of CaMKII by means of phosphatase 1 and phosphatase 2 [21]. This mechanism could act as a memory of a previous calcium impulse and play an important role in some brain functions such as memory and learning.

Some isoforms of CaMKII contain a nuclear localization sequence (NLS) that interacts with a molecule called importin. This protein is able to carry the enzyme to the nucleus, where it acts as a transcription mediator.

### 2.3. CaMKIV

The activation of CaMKIV is based on three steps. First, Ca^2+^/CaM must bind CaMKIV, which exposes threonine 196 in the activation loop, giving the enzyme a fairly weak kinase activity, which is nevertheless sufficient to induce the second activation event, autophosphorylation, in a series of serine residues at the *N*-terminal end of the protein. Finally, the kinase undergoes further phosphorylation by a CaMKK in the threonine-196 residue and its kinase activity further increases. Thus, activated CaMKIV is independent of Ca^2+^/CaM. The inactivation of the kinase occurs by the phosphatase 2A, to which it is constitutively associated [18]. CaMKIV, unlike CaMKII, does not multimerize and exists in solution as a monomer.

## 3. CaMK Function and Location

Response element binding protein (CREB), a transcription factor, which binds to cAMP response element (CRE) containing genes in the promoter, is a nuclear target of CaMK, thus becoming a key element in the regulation of expression of many genes.

In general, CaMKIV and CaMKII are mainly localized in both the nucleus and cytoplasm [22], while CaMKI appears to be cytoplasmic [23]. Rat pituitary cells, for example, contain only CaMKII (nuclear and cytoplasmic) and CaMKIV (cytoplasmic). CaMKI is not present [24].

Both CaMKII and CaMKIV phosphorylate CREB on Ser 133 while CaMKII also phosphorylates CREB on Ser 142. As we said above, after phosphorylation induced by binding to nuclear CaMKIV, both CREB and CREB-binding protein (CBP) become able to regulate the expression of several genes [25, 26]. CaMKI, too, is capable of phosphorylating CREB in Ser 133 in vitro, but it is unlikely that it can activate this transcription factor directly, given its inability to enter the nucleus [18].

CaMKs are also involved in many important cellular functions, including the release of neurotransmitters and the regulation of gene expression. CaMKIV is the most highly expressed in a small number of tissues, and its most important function is carried out in the context of neurotransmission and differentiation. In fact, it is involved in the regulation of neuronal phenomena, such as long-term potentiation (LTP) and long-term depression (LTD), which are the basis of learning and memory [18]. These phenomena have been studied mainly in the hippocampus and consist of variations in synaptic responses following prolonged stimulation of the synapse, variations that may consist of enhancement or depression of the response that persists for a long time [27]. CaMKIV also has important actions on T lymphocytes, particularly on differentiation and activation through CREB. The activation of T cells, in fact, is a multistage process that begins with the activation of the T cell receptor (TCR) and proceeds through a cascade of intracellular events that end with the production of cytokines and with the clonal expansion of the activated T cell. This complex process requires the integration of numerous signal pathways, including those dependent on the Ca^2+^/CaM complex and phosphokinase C (PKC), for the coordination of the expression of the various cytokines particularly interleukin 2 (IL2). The production of IL2 requires the activation of various transcription factors, including fos and jun, and it has been hypothesized that CREB is responsible, in this process, for the induction of both factors. CaMKII is involved in multiple cellular functions, both tissue-specific, such as the release of neurotransmitters in the synapse, and general, such as cell proliferation, secretion, and cell cycle control [18]. It also exerts direct control over cell proliferation via the Ras signal.

### 3.1. Ca^2+^/CaMK in Pineal Gland

The main activity of the pineal gland is linked with the concentration of melatonin in the blood, which is the highest in the dark and the lowest in the light. The two pineal cell types are pinealocytes, which are large, neuron-like cells and glial cells, which are astrocyte-like. They are provided with intertwined, dilated terminal processes that contain monoamines and polypeptide hormones. The gland has been shown to contain melatonin, serotonin, and norepinephrine. Calcium and magnesium salts accumulate within the pinealocytes. Serotonin-N-acetyltransferase allows the conversion of serotonin into melatonin, which seems to regulate cycles of the hypothalamus, pituitary, and gonads [28]. Ca^2+^/CaMK has been shown recently to be involved in some interesting novel pinealocyte functions. Dendrite formation has been investigated in the organotypic culture of rat hippocampus through activation of Ca^2+^/CaMKII. It seems that melatonin-activated Ca^2+^/CaMKII elicits dendritogenesis through the activation of PKC and G-protein-coupled melatonin receptors-1 and -2, thus supporting the possibility that administration of CaM and CaMKII could repair damage by dendritogenesis [29].

### 3.2. Somatolactin and Ca^2+^/CaMK Cascade in Fish Anterior Pituitary

**Figure 1:**
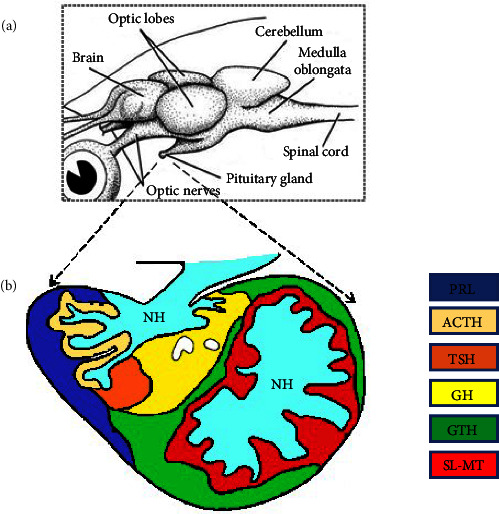
Anatomy and histology of fish pituitary. Schematic representation of fish brain showing the anatomical localization of the pituitary gland (a) and the distribution of adenohypophyseal cells in *Diplodus sargus* (b). NH, neurohypophysis (light blue). PRL, lactotropes (blue). ACTH, corticotropes (ivory white). GH, somatotropes (yellow). GTH, gonadotropes (green). TSH, thyrotropes, (orange). SL + MSH, somatolactotropes and melanotropes (red). Modified drawing from the study of Segura-Noguera et al. [30] (b) and from Living Ocean, CRDG, University of Hawaii at Manoa (a).

The fish pituitary gland is connected to the hypothalamus, which is the ventral side of the brain. A short infundibular stalk joins it to the pituitary with its pars nervosa and the brain with the hypothalamus (Figure 1).

In the Nile *Tilapia*, the activation of calcium/CaMKII through the adenylate cyclase/cyclic adenosine monophosphate/protein kinase A (AC/cAMP/PKA) and phospholipase C (PLC)/IP3/Ca^2+^/CaMKII cascade induces glucagon b, but not its isoform glucagon a, in the pituitary to stimulate the somatolactin hormone [31]. Somatolactin (SL), the latest member of the growth hormone (GH)/prolactin (PRL) family, can be identified in fish but not in higher vertebrates. Phylogenetic analysis reveals that SL is derived from ancestral GH. How glucagon affects pituitary hormone secretion is not well defined, but interestingly, the Nile *Tilapia* model helps in establishing that glucagon b acts at the pituitary level to stimulate SL release through the AC/cAMP/PKA and PLC/IP3/Ca^2+^/CaM/CaMKII cascades. Similarly, in the grass carp pituitary, the activation of neuropeptide pituitary adenylate cyclase-activating polypeptide (PACAP), through Ca^2+^/CaM-dependent mechanisms, induces SL gene expression [32], PRL promoter activation [33], PRL gene expression [34], and GH synthesis and secretion [35].

Furthermore, grass carp tachykinin-activated 3's pituitary cells could increase the synthesis and secretion of SL*α* [36], while IGF I and II administration potentiated tachykinin 3 gene product-induced SL*α* mRNA expression through calcium/CaMKII cascades.

Thus, the novel pituitary hormone SL can be mediated mainly by CaMKII although the authors of references [31–36] do not mention at what the stage of the life cycle their studies were conducted. Further investigation of the physiological importance of the link between SL and CaMKII would be worthwhile.

### 3.3. CaMKs in the Mammal Pituitary Gland

CaMKs are much more prevalent in the pituitary gland than in the pineal gland. The pituitary gland is controlled by the hypothalamus, being suspended from a portion of the third ventricle. Many aspects of the Ca^2+^/CaMK cascade, including Ca^2+^/CaMKII, are still to be clarified. As a tool to investigate the role of CaMKII, several inhibitors such as isoquinoline sulfonamide (KN-62) and similar ones have been used in intact pituitary cells. They have been found to inhibit a wide range of physiological processes in the endocrine part of the pituitary gland. It has been established that KN-62 inhibits prolactin secretion in highly KCl-stimulated pituitary cells [37].

In the rat anterior pituitary, the role of the CaMK cascade in mediating TRH-stimulated transcription of TSH and PRL has been investigated. The results indicate that the CaMKK/CaMK cascade might play an important role in TRH induction of TSH and PRL transcriptional activity in pituitary cells [38]. In addition, Altobelli et al. [24] have reported that the expression of Ca^2+^/CaMK correlated with the role of CREB in the genetic regulation of TSH. Furthermore, following TRH stimulation, CaMKIV was activated and consequently CREB was phosphorylated. The phosphorylation was linked to thyrotropin production.

However, type II and type IV CaMKs were also shown in *α*T3-1, LbetaT2 (gonadotropes), and GH3 (somatotropes) [39], as well as in normal human pituitary and pituitary adenoma, with CaMKIV being more expressed in gonadotropin-secreting tumours and CaMKII expressed in all tumour types but poorly in growth-hormone secreting tumours. These data may support the hypothesis that the presence of specific CaMK isoforms in anterior pituitary cells is linked to a specific role of pituitary cells and perhaps other endocrine tissues (this needs further studies). In addition, according to Jefferson et al. [40], multifunctional Ca^2+^/CaMK was involved in the release of prolactin in GH3 cells after stimulation with TRH, which was originally thought to be able to stimulate the activation of PKC only. The regulation of the prolactin gene by CaMKs was studied by inhibiting the gene with KN62, which limits the ability of TRH to activate the prolactin promoter [41]. It has been suggested in fact that multiple factor binding sites are necessary in the proximal and distal regions of the prolactin promoter in order to make it CaMKII sensitive.

Interestingly, while several roles for CaMKII and CaMKIV have been associated with specific anterior pituitary cytotypes, this is not the case for CaMKV, which has been localized in many organs including the rat brain and anterior pituitary [42], although it has a well-defined role in calcium regulated processes.

### 3.4. CaMKs and Hypothalamus

The hypothalamus, which controls several body functions, connects the nervous system to the endocrine system by way of the pituitary gland. It produces some hormones that through the posterior pituitary gland are directly released into the bloodstream, and some other stimulating and inhibiting hormones. The last ones reach the anterior pituitary gland and modulate the release of hormones that regulate other endocrine glands.

GRH produced by hypothalamic neurons travels to the pituitary via the hypothalamohypophyseal portal system; it exerts control over the gonadotropes of the anterior pituitary to release FSH and LH. The role of Ca^2+^/CaMK in GRH-stimulated gonadotropes in dispersed goldfish pituitary cells has been pointed out [43].

In the mouse, gonadotrope-derived LbetaT2 cells were used to show the role of Ca^2+^/CaMK in the transmission of GRH from the cell membrane to the genes of the LH subunit [44]. Ca^2+^/CaMKII, in fact, is activated by calcium in the GRH regulation of LH subunit gene transcription. Activated GRH receptor stimulates the gonadotropes of the anterior pituitary by activating the extracellular signal-regulated protein kinase (ERK) through the proline-rich tyrosine kinase 2 (Pyk2) pathway. Furthermore, it has been shown that the inhibition of CaMKII with KN93 or the knockdown of CaMKII can stop the GRH signaling pathway [45]. Interestingly, pulsatile GRH secretion can regulate FSH and LH genes differentially via several signal-regulated kinases (MAPK3, ERK), but not by CaMKII [46]. However, these observations still need clarification: Haisenleder et al. [47] stated in their report that GRH activates CaMKII but not in a frequency-dependent manner.

Some studies using transgenic mice expressing green fluorescent protein (GFP) under the promoter control for CaMKII*α* confirm high expression and the distribution of this enzyme in the brain: this expression was previously observed by immunohistochemical and biochemical studies [48–50]. Further immunohistochemical studies examined CaMKII immunoreactivity in rat hypothalamus, demonstrating a different distribution in several nuclei with different immunolabelling both between nuclei and within nuclei. Such distribution in neuronal populations may reveal differences in calcium signaling pathways [51]. In the hypothalamus, stronger GFP signals were revealed in the neuropil than in the soma of most regions [52]. It has also been reported that in GT1-7 cells (hypothalamic neuronal cell line), CaMKII*δ* regulated GRH-induced activation of MAP kinase; this being inhibited if CaMKII was inhibited with KN93, while it increased if CaMKII was overexpressed [53]. This group then found that in the same cells, after GRH treatment, PyK2 was activated. Inhibition of CaMKII*δ* with knockdown or KN93 treatment blocked the PyK2 activation, suggesting that CaMKII regulates the ERK pathway through Pyk2 activation [45]. CaMKII together with ERK1/2 and PI3K is involved in regulating the gene expression of arginine vasopressin (AVP), a hormone produced in magnocellular neurons of the hypothalamic supraoptic and paraventricular nuclei (SON and PVN, respectively). In particular, when acute slices of the rat PVN were stimulated with glutamatergic agonists that activated *N*-methyl-D-aspartate (NMDA) receptors, there was an increase in AVP gene expression. This activity is mediated by CaMKII, ERK1/2, and PI3K signal pathways [54].

Recently, murine LbetaT2 gonadotropes expressing glucocorticoid receptors have been used to show that glucocorticoids may rapidly stop LH release after GRH signaling during stress. This has also been shown in mouse pituitary explants [55]. Thus, CaMKII is involved in interactions between glucocorticoid (GC) hormones and GRH. The phosphorylation of CaMKII and synapsin-1 after stimulation in LbetaT2 and in mouse pituitary explants was meaningful, showing that membrane GC receptors and activating CaMKII interfere with the GRH pathway and with LH secretion. Interestingly, the use of Cort-bovine serum albumin, a membrane-impermeable corticosterone conjugate, and an inhibitor of protein palmitoylation (2-Br), demonstrated that GCs induce rapid signaling in LbetaT2 cells through the activation of a palmitoylated membrane GC receptor and that GCs interfere with GRH-induced calcium/CaMKII phosphorylation in LbetaT2 cells, ultimately decreasing LH release by pituitary explants. These data showed for the first time that Ca^2+^/CaMK activation plays an important role in the transmission of GRH signals from the plasma membrane to the LH subunit genes. A two-to-threefold increase in activated calcium/CaMKII was obtained after stimulation with GRH, while there was no effect after stimulation through estradiol and testosterone alone. In contrast, the combination of GRH and testosterone stimulated a threefold increase in ERK activity, thus showing that the ERK pathway and not that of Ca^2+^/CaMKII is selectively used for the regulation of FSH beta transcription by testosterone [44].

Several reports showed that endothelins (ETs), peptides of 21 amino acids with a strong vasoconstrictive effect, are expressed in some regions of the CNS including the hypothalamus functioning as neurotransmitters and/or neuropeptides [56, 57]. It has been shown that ETs are able to modulate short- and long-term regulation of tyrosine hydroxylase in rat hypothalamus through an atypical ET or ET receptor coupled to several pathways, including that of CaMKII [58, 59].

CaMKII is a signal molecule that is affected by physiological and behavioral effects due to angiotensin II (ANG II). It is established that ANG II increases the CaMKII activity in neuronal cocultures of hypothalamus and brainstem from newborn rats and that CaMKII with PKC is involved in reduction of neuronal potassium (K+) currents mediated by ANG II type 1 (AT1) receptors [60, 61]. Using the same neuronal cultures, it was shown that ANGII induces AT1 receptor-mediated chronotropic action via activation of PKC and CaMKII [62]. *In vivo* studies on rats confirmed previous *in vitro* studies. The injection of ANG II in the lateral cerebroventricle increased the dipsogenic response and CaMKII levels in the hypothalamus. Treatment with the CaMKII inhibitor reduced water intake [63].

Both CaMKIV and CaMKII have been involved in the production of hypothalamic hormones, such as corticotropin-releasing hormone (CRH). The regulation of CRH gene expression is thought to be mediated by the calcium/CaMKIV signaling pathway [64].

Several studies have shown the involvement of CaMKII in activation of light-induced Per1 gene expression resetting the biological clock in the suprachiasmatic nucleus (SCN) of hamster. In subsequent studies, the same group demonstrated by immunoblot and immunohistochemistry that the isoform CaMKII*δ* was the most expressed in the lateral ventral region neurons of the rat SCN and that it was a key regulator in Per1 expression [65, 66].

Furthermore, it has been reported that in rodents CaM and CaMKII through CREB phosphorylation are involved in circadian rhythms controlled by SCN [67]. After treatment with inhibitors of both proteins, followed by stimulation with light, an attenuation of glutamate-induced phase shift was shown suggesting their involvement in the entrainment mechanism in the SCN [68].

Hence, it would be interesting to know more about the biological significance of the distribution and possible colocalization of CaMKs throughout life in the adult hypothalamus and also during development of the brain.

### 3.5. Inhibitors of CaMK Function

Studies have demonstrated that NPY-induced feeding is regulated by CRE binding transcription factors in the rat hypothalamus and that CaMKII regulates NPY activity through CREB phosphorylation [69]. It has also been demonstrated that pCaMKII is involved in the regulation of nociception. After intratracheal injection with substance P, increased pCaMKII levels were shown using immunoblot and immunohistochemical assays in the PVN of the hypothalamus. In addition, pretreatment with the CaMKII inhibitor KN-93 confirmed the involvement of this kinase since the nociceptive response was attenuated in a dose-dependent manner [70]. However, glutamate may also inhibit CaMKII activity as has been shown by several studies describing its influence on CaMKII, particularly at high concentrations [71, 72]. In addition, exposing cultures of astrocytes derived from the preoptic area of neonatal male and female rats to high concentrations of glutamate resulted, in the males, in a prominent decrease in several activities such as cell number, cell cycle progression and CaMKII activity [73].

The intracerebroventricular administration of leptin does not show effects on CaMKII activity in the hypothalamic arcuate nucleus, while the expression of calcineurin, a CaM-dependent Ser/Thr phosphatase, increases in the same conditions [74].

## 4. Conclusion

Ca^2+^/CaMKs mediate crucial events in signal transduction activated by increased/decreased levels of intracellular Ca^2+^. In this review, we focus on signaling pathways of CaMKs in the hypothalamus, pituitary, and pineal glands. CaM kinases, which are highly expressed in brain and other tissues, have also been found in brain endocrine cells specialized for the secretion of hormones that ensure whole body homeostasis. The CaMKs have different locations, suggesting that they are involved in various physiological conditions and cell functions. Knowledge of the molecular mechanisms regulated by these ubiquitous enzymes is important for understanding the physiology and the dysfunction of endocrine cells in the brain and glands. Indeed, CaMK inhibitors are used in many cancer therapies. They are known to cause adverse side effects associated with the endocrine system [75]. These endocrine side effects and the consequences associated with these drugs should be clarified in the endocrine brain. Here, even if CaMKs have been widely studied in other fields, the data dealing with their roles are still restricted. The reviewed literature suggests that the CaMK families are worthy of study and are of considerable interest for the impact that these signal molecules may have on several aspects of the brain's endocrine glands. Thus, although the key data in this review are by no means exhaustive, the importance of various endogenous calcium binding proteins is highlighted.

## Figures and Tables

**Table 1:** Distribution of CaMKs in cell compartments. The confidence values for each cell compartment are indicated. The highest confidence for proteins of multifunctional CaMKs is in cytosol and nucleus, which are therefore assumed to be the subcellular compartments richest in CaMKs. With the exception of endoplasmic reticulum reaching confidence value 4 for CaMII*β*, CaMKII*γ,* and CaMK*δ*, the other organelles are at level 1, the lowest confidence level. Confidence values of cellular compartments are obtained from the GeneCards-the human gene database (https://www.genecards.org/) [11].

Compartments	CaMKI	CaMKII*α*	CaMKII*β*	CaMKII*γ*	CaMKII*δ*	CaMKIV
Cytosol	5	5	5	5	5	4
Nucleus	4	5	4	4	5	5
Extracellular	1	2	2	1	1	4
Mitochondrion	2	4	2	1	1	3
Cytoskeleton	2	2	4	1	2	2
Lysosome	1	1	1	1	1	1
Plasma membrane	2	2	2	2	4	2
Endoplasmic reticulum	1	2	4	4	4	2
Endosome	1	1	1	1	1	1
Peroxisome	1	0	—	1	1	1
Golgi apparatus	1	1	1	1	1	1
